# The Predictive Role of the Triglyceride/Glucose Index in Patients with Hypercholesterolemia and Acute Ischemic Stroke

**DOI:** 10.31083/j.rcm2312399

**Published:** 2022-12-09

**Authors:** Christodoula Kourtidou, Eleftheria Ztriva, Danai-Thomais Kostourou, Georgios Polychronopoulos, Sarantis Satsoglou, Georgios Chatzopoulos, Anastasia Kontana, Marios Tzavelas, Evripidis Valanikas, Stavroula Veneti, Areti Sofogianni, Dimitrios Milonas, Achilleas Papagiannis, Christos Savopoulos, Konstantinos Tziomalos

**Affiliations:** ^1^First Propedeutic Department of Internal Medicine, Medical School, Aristotle University of Thessaloniki, AHEPA Hospital, 54636 Thessaloniki, Greece

**Keywords:** ischemic stroke, severity, outcome, triglyceride/glucose index, insulin resistance, prognosis, hypercholesterolemia

## Abstract

**Background:**

The triglyceride/glucose index (TyG) reflects insulin resistance and predicts the risk of acute ischemic stroke (aIS). However, it is uncertain if this index predicts the severity and outcome of aIS because studies that addressed this question are few and all were performed in Asian subjects. Moreover, there are no studies that focused on patients with hypercholesterolemia.

**Methods:**

We studied 997 Caucasian patients who were hospitalized for aIS and had hypercholesterolemia. aIS severity was assessed at admission with the National Institutes of Health Stroke Scale (NIHSS) and severe aIS was defined as NIHSS ≥21. The outcome was assessed with the functional outcome at discharge and with in-hospital mortality. An unfavorable functional outcome was defined as modified Rank in scale (mRs) at discharge between 3 and 6.

**Results:**

The TyG index did not correlate with the NIHSS at admission (r = 0.032, *p* = NS) and was similar in patients with severe and non-severe aIS (8.7 ± 0.6 and 8.6 ± 0.6, respectively; *p* = NS). Risk factors for severe aIS were age, female gender, atrial fibrillation (AF) and diastolic blood pressure (DBP) at admission. The TyG index also did not correlate with the mRs(r = 0.037, *p* = NS) and was similar in patients who had unfavorable and favorable functional outcome (8.7 ± 0.6 and 8.6 ± 0.5, respectively; *p* = NS). Risk factors for unfavorable functional outcome were age, previous ischemic stroke, body mass index and the NIHSS at admission. The TyG index was similar in patients who died during hospitalization and patients who were discharged (8.7 ± 0.6 and 8.7 ± 0.6, respectively; *p* = NS). Risk factors for in-hospital mortality were AF and DBP and NIHSS at admission.

**Conclusions:**

The TyG index does not appear to be associated with the severity or the outcome of aIS. Nevertheless, since there are few relevant data in Caucasians and the TyG index is an inexpensive and widely available biomarker, more studies in this ethnic group are required to determine the predictive role of this index in patients with aIS.

## 1.Introduction

Ischemic stroke is a leading cause of long-term disability and mortality worldwide [1, 2]. Stroke risk stratification is essential for reducing stroke-relatedd eath and disability [3]. The prediction of functional outcome of patients who experience an acute ischemic stroke (aIS) is another crucial component of the management of this population [4, 5]. Several predictive models of stroke outcome have been developed but have suboptimal accuracy [6, 7]. Accordingly, development of novel prognostic factors is needed to identify patients at increased risk for severe stroke and adverse outcome [8, 9].

Several studies showed that the triglyceride-glucose (TyG) index is an accurate marker of insulin resistance (IR), particularly in South American and Asian populations [10, 11]. Indeed, the TyG index has comparable sensitivity and specificity with the euglycemic hyperinsulinemic clamp, which is the gold standard test for determining IR [10]. Moreover, the TyG index is a low-cost, simple and universally available marker [10, 11]. On the other hand, a study in non-diabetic overweight or obese postmenopausal Caucasian women reported that the TyG index correlates relatively modestly with the euglycemic hyperinsulinemic clamp, suggesting that the TyG index may not perform equally well in all populations [12]. Given the role of IR in the pathogenesis of atherosclerosis, several prospective studies evaluated the association between the TyG index and the incidence of ischemic stroke in the general population and showed that this index predicts stroke risk [13, 14, 15]. However, whether the TyG index predicts the severity and outcome of aIS is less clear because studies that addressed this question are few and all were performed in Asian subjects [16, 17, 18, 19]. Moreover, there are no studies that assessed this association specifically in patients with aIS and hypercholesterolemia, which is a major risk factor for ischemic stroke.

The aim of the present study was to assess the relationship between the TyG index and aIS severity and outcome in Caucasian patients with hypercholesterolemia admitted with aIS [20].

## 2. Patients and Methods

All patients who were admitted with aIS between 9/2010 and 2/2018 in our department were prospectively enrolled in the present study (n = 1107). Patients were enrolled consecutively and no patient with aIS who was admitted during this time period was excluded from the study. All patients were followed-up until discharge.

At admission, a physical examination was performed and a detailed medical history including the presence of cardiovascular risk factors and established cardiovascular disease was obtained. Stroke severity was determined with the National Institutes of Health Stroke Scale (NIHSS). Severe stroke was defined as NIHSS ≥21.

At the first day after admission, routine biochemistry testing was performed, including serum levels of glucose, lipids, creatinine and uric acid. The TyG index was calculated using the following formula: TyG = Ln [fasting triglyceride (mg/dL) × fasting glucose (mg/dL)/2] [10]. Hypercholesterolemia was defined as the use of lipid-lowering agents at admission or LDL-C levels >55 mg/dL, according to the current targets for patients with ischemic stroke [20]. The glomerular filtration rate was estimated with the CKD-EPI equation [21].

The outcome was assessed with the functional outcome at discharge and with in-hospital mortality. An unfavorable functional outcome was defined as modified Rankin scale at discharge between 3 and 6.

The study was approved by the Ethics Committee of AHEPA Hospital, Thessaloniki, Greece. All patients provided written informed consent.

## 3. Statistical Analysis

All data were analyzed with IBM SPSS Statistics for Windows (version 27, IBM Corp., Armonk, NY, USA). The chi-square test was used to detect differences in categorical variables between groups. The independent samples *t*-test was applied to identify differences in continuous variables between groups. Spearman’s correlation was used to identify correlations between continuous variables. Binary logistic regression analysis was applied to detect variables independently associated with severe stroke, unfavorable functional outcomeand in-hospital death.

## 4. Results

At admission, 997 patients had hypercholesterolemia and their characteristics are shown in Table 1. At admission, 13.0% of patients had severe stroke (n = 130). The TyG index did not correlate with the NIHSS (r = 0.032, *p* = NS) and was similar in patients with severe and non-severe stroke (8.7 ± 0.6 and 8.6 ± 0.6, respectively; *p* = NS; odds ratio (OR) 1.083, 95% confidence interval (CI) 0.774–1.515, *p* = NS). Patients with severe stroke were older, less frequently males and smokers, and had greater prevalence of atrial fibrillation (AF) and higher diastolic blood pressure (DBP) (Table 2). Risk factors for severe stroke were age (OR 1.078, 95% CI 1.042–1.114, *p *< 0.001), female gender (OR 1.827, 95% CI 1.162–2.870, *p *< 0.01), AF (OR 1.959, 95% CI 1.289–2.979, *p *< 0.005) and DBP (OR 1.019, 95% CI 1.005–1.032, *p *< 0.01).

**Table 1.** **Patients’ characteristics at admission (n = 997)**.

Age (years)	79.8 ± 7.2
Males (%)	41.7
Systolic blood pressure (mmHg)	151 ± 27
Diastolic blood pressure (mmHg)	81 ± 15
Heart rate	78 ± 15
Hypertension (%)	80.7
Type 2 diabetes mellitus (%)	32.6
Type 2 diabetes mellitus duration (years)	12.3 ± 8.3
Smoking (current/past, %)	14.0/14.7
Package-years	71 ± 51
Atrial fibrillation (%)	36.4
Body mass index (${\mathrm{kg/m}}^{2}$)	27.6 ± 5.1
Waist circumference (cm)	104 ± 12
Waist/hip	0.98 ± 0.07
Overweight/obese (%)	40.8/26.8
Family history of cardiovascular disease (%)	17.0
Coronary heart disease (%)	25.8
Prior ischemic stroke (%)	44.5
Heart failure (%)	16.0
Glucose (mg/dL)	116 ± 49
Low-density lipoprotein cholesterol (mg/dL)	109 ± 37
High-density lipoprotein cholesterol (mg/dL)	47 ± 14
Triglycerides (mg/dL)	117 ± 54
Triglyceride glucose index	8.7 ± 0.6
Uric acid (mg/dL)	5.8 ± 1.9
Estimated glomerular filtration rate (mL/min/1.73 $m^{2}$)	64 ± 20
National Institutes of Health Stroke Scale	9.0 ± 8.9

**Table 2.** **Differences between patients with severe stroke at admission (National Institutes of Health Stroke Scale (NIHSS) at admission ≥21) and those with non-severe stroke (NIHSS <21)**.

	Patientswithsevere stroke	Patientswith non-severe stroke	*p*
	(n = 130)	(n = 867)	
Age (years)	82.6 ± 7.6	79.3 ± 7.0	<0.001
Males (%)	30.8	43.9	<0.01
Diastolic blood pressure (mmHg)	84 ± 17	81 ± 14	<0.05
Smoking (current/past, %)	10.8/8.5	15.2/15.7	<0.05
Atrial fibrillation (%)	54.6	34.1	<0.001

At discharge, 57.9% of patients had unfavorable functional outcome (n = 577). The TyG index did not correlate with the modified Rank in scale (r = 0.037, *p* = NS) and was similar in patients with unfavorable and favorable functional outcome (8.7 ± 0.6 and 8.6 ± 0.5, respectively; *p* = NS). Patients who had unfavorable functional outcome were older, less frequently males and smokers, and had greater prevalence of AF, previous ischemic stroke and family history of CVD, higher heart rate and body mass index (BMI), lower eGFR and higher NIHSS (Table 3). Risk factors for unfavorable functional outcome were age (OR 1.069, 95% CI 1.032–1.108, *p *< 0.001), previous ischemic stroke (OR 1.717, 95% CI 1.083–2.723, *p *< 0.05), BMI (OR 1.051, 95% CI 1.001–1.103, *p *< 0.05)and NIHSS (OR 1.418, 95% CI 1.327–1.488, *p *< 0.001).

**Table 3.** **Differences between patients with unfavorable functional outcome at discharge (modified Rankin scale 3–6) and those with favorable functional outcome at discharge (modified Rankin scale 0–2)**.

	Patientswith unfavorablefunctional outcomeat discharge	Patientswith favorablefunctional outcomeat discharge	*p*
	(n = 577)	(n = 420)	
Age (years)	81.2 ± 7.1	77.6 ± 6.9	<0.001
Male gender (%)	36.2	48.8	<0.001
Heart rate	79 ± 15	77 ± 15	<0.05
Smoking (current/past, %)	13.2/11.4	15.0/20.2	<0.001
Atrial fibrillation (%)	42.1	29.0	<0.001
Body mass index (${\mathrm{kg/m}}^{2}$)	28.0 ± 5.4	27.2 ± 4.7	<0.05
Family history of cardiovascular disease (%)	20.1	13.8	<0.05
Prior ischemic stroke (%)	49.9	39.3	<0.005
Estimated glomerular filtration rate (mL/min/1.73 $m^{2}$)	62 ± 21	66 ± 19	<0.005
National Institutes of Health Stroke Scale	13.2 ± 9.1	2.9 ± 3.2	<0.001

During hospitalization, 9.3% of patients died (n = 93). The TyG index was similar in patients who died and patients who were discharged (8.7 ± 0.6 and 8.7 ± 0.6, respectively; *p* = NS; OR 1.095, 95% CI 0.756–1.586, *p* = NS). The former were older and had greater prevalence of AF, higher systolic and diastolic BP and heart rate, higher uric acid levels, and higher NIHSS (Table 4). Risk factors for death were AF (OR 2.188, 95% CI 1.178–4.063, *p *< 0.05), DBP (OR 1.044, 95% CI 1.023–1.065, *p *< 0.001) and NIHSS (OR 1.190, 95% CI 1.151–1.231, *p *< 0.001).

**Table 4.** **Differences between patients who died during hospitalization and those who were discharged**.

	Patientswho died duringhospitalization	Patientswho were discharged	*p*
	(n = 93)	(n = 904)	
Age (years)	82.9 ± 7.3	79.3 ± 7.1	<0.001
Systolic blood pressure (mmHg)	157 ± 31	150 ± 26	<0.05
Diastolic blood pressure (mmHg)	88 ± 18	80 ± 14	<0.001
Heart rate	83 ± 16	78 ± 15	<0.001
Atrial fibrillation (%)	63.4	33.2	<0.001
Uric acid (mg/dL)	6.3 ± 2.0	5.7 ± 1.8	<0.01
National Institutes of Health Stroke Scale	23.7 ± 8.5	7.4 ± 7.3	<0.001

We performed subgroup analyses to evaluate the association between the TyG index and stroke severity, functional outcome at discharge and in-hospital mortality in patients with normal and abnormal cholesterol levels, in patients treated and not treated with lipid-lowering agents, and in patients with type 2 diabetes mellitus. In all these subgroups, the TyG index did not predict stroke severity, functional outcome at discharge or in-hospital mortality.

## 5. Discussion

A principal finding of our study is that the TyG index is not related with the severity of ischemic stroke at admission. This is the first study that assessed the relationship between this index and aIS severity in Caucasian patients. In 2 previous studies from China and Taiwan, the NIHSS at admission did not differ across TyG quartiles or between patients with TyG <8.4 or ≥8.4 [19, 22]. However, the authors did not perform multivariate analyses to determine whether the TyG index is independently associated with stroke severity [19, 22]. In contrast, in a prospective study in 1226 non-diabetic Chinese patients with aIS, the NIHSS at admission was paradoxically lower in patients with high TyG index [23]. However, multivariate analysis to identify an independent association between this indexand stroke severity was again not performed [23]. Given the methodological limitations of these studies and that they were all performed in Asian subjects [19, 22, 23], more data are needed to clarify whether the TyG index predicts stroke severity.

In our cohort, the TyG index did not predict the functional outcome. In agreement with our observation, a large study performed in China (n = 16,310 patients with ischemic stroke) also showed that the TyG index does not predict the functional outcome at 12 months after stroke [19]. In contrast, small, retrospective studies conducted in South Korea, Taiwan or Singapore reported an independent association between a high TyG index and poor functional outcome at 3 months after discharge [16, 17, 24]. However, the retrospective design of most of the latter studies represents a substantial source of bias [16, 17, 24]. Moreover, these studies included selected subgroups of patients with aIS, i.e., either patients who underwent reperfusion therapy [16, 24] or patients with a single subcortical infarction [17]. Paradoxically, a large prospective study in 12,964 non-diabetic Chinese patients with aIS reported that a high TyG index predicts favorable functional outcome at 12 months after stroke [24]. It is possible that the inclusion of non-diabetic patients might partly explain this finding [24]. Another potential reason for these contrasting findings is that the TyG index is a less accurate marker of IR in Caucasians, as shown in studies comparing this marker of IR with the euglycemic hyperinsulinemic clamp [10, 11, 12].

In our study, the TyG index did not predict mortality during hospitalization. There are few data about the usefulness of the TyG index for predicting in-hospital mortality in patients with aIS. In a large retrospective study (n = 4570), a high TyG index predicted hospital and intensive care unit mortality in Chinese patients with ischemic stroke [18]. However, the aforementioned study included only critically ill patients, limiting the generalizability of the results [18]. Studies in Asian patients with ischemic stroke also reported an independent relationship between the TyG index and all-cause mortality at 90 days [25] or at 12 months after stroke [19, 23]. In contrast, other studies from China and Taiwan did not identify a relationship between this index and all-cause mortality at 90 days or 12 months after stroke [22, 24]. The longer follow-up applied in the latter studies might capture different causes of death than the evaluation of in-hospital mortality. In addition, the conflicting findings, even in patients of the same ethnicity, highlight the need for further research in the field.

Our study has several limitations. First, we did not consider all relevant risk factors for ischemic stroke, particularly obstructive sleep apnea, and this represents a weakness of our study. Indeed, several studies showed that obstructive sleep apnea (OSA) is associated with increased risk for ischemic stroke [26, 27]. Moreover, patients with OSA appear to have worse functional outcome after ischemic stroke [28]. It appears that these associations are partly independent and partly due to the relationship between OSA and hypertension, atrial fibrillation and obesity [29, 30]. In turn, obesity is strongly related to IR and is also associated with increased risk for ischemic stroke [31, 32]. Second, it is possible that TyG index might have been more useful for the prediction of stroke severity if it was determined prior to stroke.

## 6. Conclusions

Our study suggests that the TyG index is not associated with the severity of aIS at admission or the outcome of these patients. However, given the paucity of data on this topic in Caucasians and the low cost and wide availability of the TyG index, more studies in this ethnic group are required to assess the predictive value of this index in aIS.

## Data Availability

All datasets used during the current study are available from the senior author upon reasonable request.
